# Pharmacokinetic Herb-Drug Interaction between *Hibiscus sabdariffa* Calyces Aqueous Extract and Captopril in Rats

**DOI:** 10.1155/2020/5013898

**Published:** 2020-06-17

**Authors:** Shinta Ayu Nurfaradilla, Fadlina Chany Saputri, Yahdiana Harahap

**Affiliations:** ^1^Graduate Program, Faculty of Pharmacy, Universitas Indonesia, Kampus UI, Depok 16424, Indonesia; ^2^Laboratory of Pharmacology, Faculty of Pharmacy, Universitas Indonesia, Kampus UI, Depok 16424, Indonesia; ^3^Laboratory of Bioavailability and Bioequivalency, Faculty of Pharmacy, Universitas Indonesia, Kampus UI, Depok 16424, Indonesia

## Abstract

*Hibiscus sabdariffa* L. (Malvaceae) is a traditional medicinal herb widely consumed as a beverage (“hibiscus tea”), and its global popularity is expanding due to health benefits such as blood pressure and cholesterol control. Previous studies showed that *Hibiscus sabdariffa* is coadministered with antihypertensives and antihyperlipidemics, thus predisposing herb-drug interactions. We investigated the pharmacokinetic interaction between *H. sabdariffa* L. aqueous extract and captopril, a frequently prescribed antihypertensive. In this study, chemical profile of *H. sabdariffa* L. aqueous extract was identified using HPLC system equipped with a DAD detector at 360 nm and 520 nm. The male Sprague Dawley rats were divided into two groups of six rats. Group I received a single dose of captopril suspension (4.5 mg/200 g body weight (BW) orally (p.o.)) while group II received *H. sabdariffa* L. aqueous extract (60 mg/200 g BW; p.o.) daily for two weeks prior to the same captopril dose. Multiple blood samples were collected at predetermined times after captopril administration and the plasma concentration was analyzed using ultrahigh-pressure liquid chromatography-tandem mass spectrometry. Chemical profiling of the *H. sabdariffa* L. aqueous extract showed that the extract contains chlorogenic acid, myricetin 3-arabinogalactoside, 5-O-caffeoylshikimic acid, quercetin 3-rutinoside, delphinidin 3-sambubioside, and cyanidin 3-sambubioside. Ingestion of the extract significantly reduced the captopril area under the curve (AUC)_0−t_ (0.1745 (0.1254–0.2429)), AUC0_−∞_ (0.1734 (0.1232–0.2442))], and peak plasma concentration (0.2119 (0.1337–0.3359)) (geometric mean ratio of the coadministration group to the captopril group (90% CI)). The geometric mean ratios were falling outside the 90% CI of 0.8–1.25 bioequivalent range. Conversely, *H. sabdariffa* L. extract increased the apparent total body clearance (Cl/F, 0.0257 ± 0.0115 vs. 0.1418 ± 0.0338 mL/h·kg) and the apparent volume of distribution (Vd/F, 0.0541 ± 0.0226 vs. 0.3205 ± 0.0790 mL/kg). This study indicated that coadministration of *H. sabdariffa* L. aqueous extract could change the pharmacokinetic profile of captopril; therefore, its coadministration should be avoided.

## 1. Introduction

*Hibiscus sabdariffa* L. (Malvaceae) is an herbaceous shrub grown in tropical areas such as India, Malaysia, Africa, Australia, Florida, the Philippines, and Indonesia [1]. Beverages made from *H. sabdariffa* L. (variously referred to as “sour tea” or “hibiscus tea”) are consumed in many regions of the world and extracts have been used as traditional medicines in numerous cultures [2–4]. The consumption of hibiscus tea is growing in popularity owing to accumulating evidence that it can safely reduce blood pressure and serum cholesterol, two major risk factors for cardiovascular disease [3]. Furthermore, benefits against cancer, excess weight, liver disease, infertility, and diabetes have been proposed [1, 4]. A survey by Showande et al. [5] on staff and students at the University of Ibadan in Africa found that some respondents often consume sour tea with pharmaceuticals such as antibiotics, antihypertensives, antipsychotics, antihyperlipidemics, and antiretrovirals. It is therefore important to determine whether there are herb-drug interactions that may interfere with the primary drug activity. Indeed, previous preclinical and clinical studies showed that ingestion of *H. sabdariffa* L. extract with conventional pharmaceuticals such as acetaminophen, chloroquine, hydrochlorothiazide, and simvastatin can change the pharmacokinetics profile of the primary drug, potentially affecting the treatment efficacy [5–9].

Animal models, particularly rodents, are widely used in preclinical studies and have become a valuable tool for providing information on the efficacy, safety, and mechanism of action of various drugs and compounds used in treatment [10, 11]. The findings of animal research could traditionally be projected to humans [10]. One of the reasons of using animals in biomedical research is due to its similarities to human, particularly its similarity in anatomical basis and physiological functions with humans [11].

In Indonesia, *H. sabdariffa* L. is often consumed with angiotensin-converting enzyme inhibitor captopril, a frequently prescribed antihypertensive [12]; however, a previous study showed that coadministration of *H. sabdariffa* does not influence the antihypertensive potency of captopril in a 2K1C rat hypertension model [13]. To our knowledge, there is no available information regarding pharmacokinetic interactions between captopril and *H. sabdariffa.* Therefore, this study examined changes in the captopril pharmacokinetic characteristics in rats following prolonged administration of *H. sabdariffa* L. aqueous extract.

## 2. Materials and Methods

### 2.1. Chemical and Herbal Extract

Reference standard captopril, propranolol hydrochloride (used as the internal standard (IS)), and the derivatization reagent 2-4-dibromoacetophenone were purchased from Sigma-Aldrich (Singapore). HPLC grade acetonitrile (ACN) and formic acid were purchased from Merck Chemicals (Germany). The calyces of *H. sabdariffa* L were procured from the Research Institute for Medicinal and Aromatic Plants (Bogor, Indonesia) and identified by the Indonesian Institute of Sciences Center for Plant Conservation Botanic Garden (Bogor, Indonesia; reference number B-2306/IPH.3/KS/VII/2018). Extraction was performed by maceration at 50°C for 6-7 hours (ratio between simplicia and water was 1 : 6). The aqueous extract was evaporated until it forms the honey-like form and then stored at 4°C–10°C. The yield of extract was 35.4%. This procedure was performed at the Research Institute for Medicinal and Aromatic Plants. The captopril sample used for pharmacokinetic measurements was obtained from 25 mg generic tablets which were marketed in Indonesia.

### 2.2. Chemical Profile of *Hibiscus sabdariffa* Extracts

Chemical profile of *H. sabdariffa* L. aqueous extract was identified using a high-pressure performance chromatography (HPLC) system (Agilent Technologies 1200 Series HPLC-0053, Germany) equipped with a diode-array detector (DAD; serial no. DE60555816). Compounds separation was performed using a reversed phase C18 column (Inertsil ODS-3; 5.0 *µ*m, 4.6 × 150 mm; Japan) as the stationary phase and two gradient programs of the mobile phase running at a flow rate of 0.5 mL/min [14]. Gradient program 1 contained a mixture of solution A (1% formic acid : ACN (10 : 90)) and solution B (ACN) while gradient program 2 contained a mixture of solution A (10% formic acid in water) and solution B (ACN). Detections were performed at 360 nm for gradient program 1 and 520 nm for gradient program 2. The injection volume for the sample was 20 *µ*L.

### 2.3. Experimental Animals

Twelve adult male Sprague Dawley rats (approximately 3 months old) weighing 150–250 g were obtained from Bogor Agricultural University (IPB; Bogor, Indonesia). The rats were housed under a controlled ambient temperature (25 ± 5 °C) and a 12 h : 12 h light : dark cycle with free access to food and water. The bedding for each cage was replaced thrice a week. All animal care and experimental procedures were prepared according to Animal (Scientific Procedures) Act 1986 and were reviewed as well as approved by the Ethics Committee of the Faculty of Medicine, Universitas Indonesia (Jakarta, Indonesia) under the reference number 0646/UN2.F1.ETIK/2018.

### 2.4. Validation Method and Analysis of Captopril

Captopril was measured in the plasma using a modified ultrahigh-pressure liquid chromatography-tandem mass spectrometry (UPLC-MS/MS) method [15–18]. Chromatography was performed using a reversed phase C18 column (Acquity UPLC BEH Shield RP; 1.7 *µ*m, 2.1 × 100 mm; Waters, Milford, CT, USA) at a column temperature of 40°C. A mixture of 0.1% formic acid and ACN was used as the mobile phase with the gradient elution profile shown in Table 1. Captopril was detected using a Xevo TQD mass spectrometer (Waters, USA) equipped with a positive electrospray ionization ion source in multiple reaction monitoring modes. The operational parameters for mass detection are presented in Table 2. Acquisition and integration of data were performed using MassLynx 4.1 SCN805 Software solution.

The stock solution of the reference standard captopril (1000 mg/L) was prepared by dissolving 10 mg of the reference standard in 10 mL aquadest. The stock solution of propranolol (1000 mg/L, IS) was prepared by dissolving 10 mg of the reference standard in 10 mL methanol : aquadest (50 : 50). The stock solution of 2-4-dibromoacethophenone (520 mg/L) was prepared by dissolving 5.2 mg in 10 mL methanol. Quality control samples and calibration standards were prepared by diluting standard stock solutions with rat plasma. The captopril quality control samples were prepared at concentrations of 9, 40, and 80 ng/mL, while the calibration standards were prepared at concentrations of 3, 9, 12, 25, 50, 80, and 100 ng/mL.

The validation method in analysis of captopril was performed by examining the accuracy, precision, sensitivity, selectivity, matrix effect, carry over, and stability profiles of captopril according to EMA guidelines.

### 2.5. Preparation of Plasma Sample

Blood samples were collected in EDTA tubes and centrifuged at 5,000 rpm for 10 min to obtain plasma. For derivatization of plasma captopril, a plasma sample (180 *µ*L) was first mixed with 20 *µ*L of 520 ppm 2-4-dibromoacetophenone stock solution and 20 *µ*L of 5% ammonia solution, vortexed for 30 seconds, and incubated at room temperature for 30 min. The derivatization reaction was stopped by adding 20 *µ*L of 15% formic acid. Then, 20 *µ*L of 1 mg/L propranolol (IS) was added to the mixture and vortexed for 30 s [17, 18]. Plasma proteins were precipitated by adding 600 *µ*L ice-cold ACN, vortexing for 30 s, and centrifuging at 12,000 rpm for 5 min. Supernatant samples (600 *µ*L) were evaporated by nitrogen gas flowing at 60°C for 15 min. The residue was dissolved in 300 *µ*L of the mobile phase and transferred to an autosampler vial. The injection volume for UPLC-MS/MS was 5 *µ*L.

### 2.6. Pharmacokinetic Interaction Study

The experimental animals were divided randomly and blindly into two groups (*n* = 6/group). Group I received single dose captopril suspension (4.5 mg/200 g BW; p.o.) while group II received *H. sabdariffa* L. aqueous extract (60 mg/200 g BW; p.o.) daily for two weeks at morning and a single captopril dose (4.5 mg/200 g BW; p.o.) immediately after the last extract administration. The animals were anaesthetized before blood collection process. Blood samples (approximately 0.36 mL per sample) were obtained before captopril administration and 5, 15, 30, 45, 60, 120, 180, 300, 420, and 600 min after administration. The loss of body fluid was compensated by administrating 1.0 mL saline solution (p.o.) every 30 min. Plasma was then isolated from blood samples as described.

### 2.7. Data Analysis

The captopril pharmacokinetic parameters area under curve (AUC_0−t_ and AUC_0−∞_), peak plasma concentration (*C*max), time at peak plasma concentration (*T*max), half-life (T_1/2_), apparent total body clearance (Cl/F), apparent volume of distribution (Vd/F), and elimination constant (Ke) were estimated using PKSolver® compartmental analysis software. All data are expressed as mean ± standard deviation. Group means were compared by Mann–Whitney *U* test using SPSS (v24.0). A *P* < 0.05 (two-tailed) was considered statistically significant for all tests. Geometric mean ratios with 90% confidence intervals (CI) for AUC and *C*max of the coadministration group versus the captopril group were calculated. Herb-drug interaction was established if the geometric mean ratios with 90% CI for AUC and *C*max were outside of the bioequivalence range (0.8–1.25).

## 3. Results and Discussion

### 3.1. Chemical Profile of *Hibiscus sabdariffa* Extracts

**Figure 1:**
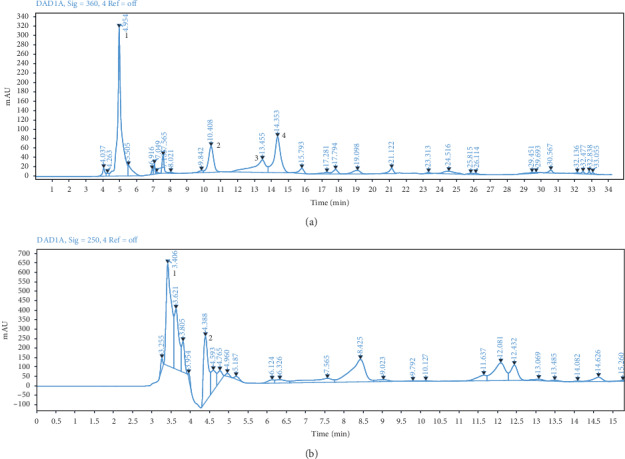
Chromatographic profile of *Hibiscus sabdariffa* L aqueous extract using the gradient programs described in methods. (a) UV chromatogram at 360 nm using gradient program 1. (b) UV chromatogram at 520 nm using gradient program 2.

Separation and identification of polyphenols, HCA, and lactone were performed using gradient program 1 at 360 nm, while anthocyanins were detected using gradient program 2 at 520 nm [13]. The chemical profile (Figure 1) showed that *H. sabdariffa* aqueous extracts contained chlorogenic acid (1), myricetin 3-arabinogalactoside (2), 5-O-caffeoylshikimic acid (3), quercetin 3-rutinoside (4), delphinidin 3-sambubioside (5), and cyanidin 3-sambubioside (6). Anthocyanins were detected using a gradient elution with 10% formic acid and detection at 520 nm since the acid-base equilibrium state of this class is stable at acidic pH [14]. Among the compounds identified, delphinidin 3-sambubioside and cyanidin 3-sambubioside have been shown to inhibit angiotensin-converting enzyme while quercetin has been shown the vasodilator activity [19, 20].

### 3.2. Experimental Animal

All rats used in this experiment were weighted prior to treatment procedure. The weights of rats were in the range of 150–250 g (172.92 ± 21.90). The rats were also in good health condition, which was shown by clear eyes, normal behaviour, and motoric movement.

### 3.3. Chromatography of Captopril

**Figure 2:**
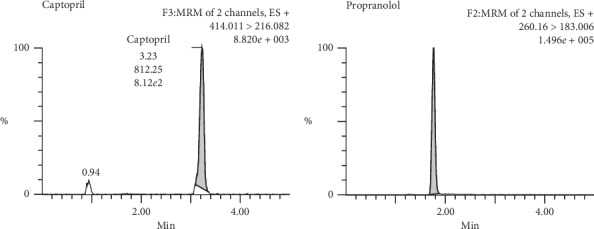
Chromatogram of derivate captopril and propranolol.

The chromatography system yielded shape peaks for propranolol and captopril derivatives, with retention times of 1.73 and 3.22 min, respectively (Figure 2).

### 3.4. Linearity, Lower Limit of Quantification (LLOQ), and Accuracy and Precision

The captopril calibration curve was linear over the range 3–100 ng/mL (*r* = 0.9998). The LLOQ was 3 ng/mL with a coefficient of variation (%CV) of 14.38% and a % Diff between −16.39% and 16.07%. The within-run accuracy and precision of captopril analysis ranged from 1.56% to 14.38% (Table 3).

### 3.5. Selectivity

In selectivity testing, six rats plasma was used. Selectivity testing was performed on LLOQ concentration and the interference value was compared to blank plasma solution. The results showed that the interference values of endogenous substances were within limit.

### 3.6. Carry over

The maximum of carry over value is 1.16% for captopril and 3.22% for propranolol. Those values were within the acceptance criteria for the analyte (<20%) and the internal standard (<5%).

### 3.7. Matrix Effect

Ion suppression was observed during matrix effect testing (0.97); however, the value is still within the acceptance criteria (0.80–1.20).

### 3.8. Stability Test

The stability profiles of captopril were assessed in different storage conditions. Based on our study, the QCH and QCL solutions were stabile when stored in room temperature for 24 hrs, in autosamples for 24 hrs, and in freezer (−25°C) for 7 days. The QCL and QCH solutions were also stabile after 3 cycles of freeze-thaw.

### 3.9. Pharmacokinetics Interaction Study

**Figure 3:**
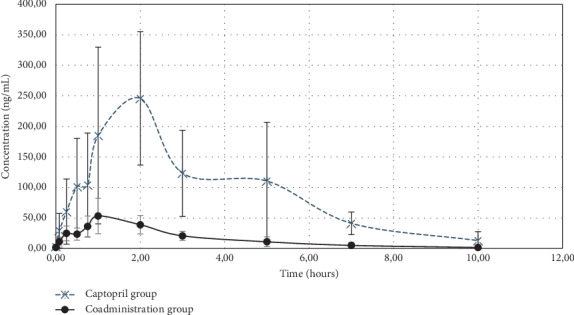
Mean plasma concentration vs. time curves of the captopril group and on coadministration with *Hibiscus sabdariffa* aqueous extract in rats.

The mean captopril plasma concentration versus time curve is shown in Figure 3 and the pharmacokinetic parameters are listed in Table 4. Consumption of *H. sabdariffa* L. aqueous extract for two weeks reduced the captopril AUC_0−t_ (956.86 ± 314.28 vs. 162.35 ± 41.00 ng·h/mL), AUC_0−∞_ (993.32 ± 339.55 vs. 166.72 ± 41.82 ng·h/mL), and *C*max (282.98 ± 124.79 vs. 60.02 ± 28.84 ng/mL) significantly (all *P* < 0.05) and increased Cl/F (0.0257 ± 0.0115 vs. 0.1418 ± 0.0338 mL/h kg) and Vd/F (0.0541 ± 0.0226 vs. 0.3205 ± 0.0790 mL/kg) significantly (both *P* < 0.05). Geometric mean ratios (90% CI) for AUC_0−t_, AUC_0−∞_, and *C*max between coadminisration and captopril groups could be seen in Table 5. The geometric mean ratio and 90% confidence intervals (CI) of the coadministration group to the captopril group were 0.1745 (0.1254–0.2429) for AUC_0−t_, 0.1734 (0.1232–0.2442) for AUC_0−∞_, and 0.2119 (0.1337–0.3359) for *C*max, respectively. Those geometric mean ratios of AUC and *C*max were not bioequivalent.

### 3.10. Effect of Coadministration of *H. sabdariffa* L Aqueous Extract on Pharmacokinetic Profile of Captopril

*H. sabdariffa* L. aqueous extract or sour tea is growing in popularity due to a variety of purposed health benefits, but several studies have reported that its consumption can alter the pharmacokinetic profiles and potential efficacies of prescribed medications [5–9]. Among the best described health benefits of *H. sabdariffa* L. is blood pressure control for hypertension, suggesting that many patients on conventional antihypertensives may also consume *H. sabdariffa* L. extract [3, 5, 12].

Administration of the extract was given two weeks prior to the administration of captopril since the extracts need time to induce the enzyme metabolizing captopril. Moreover, the study literature showed that two-week period of *H. sabdariffa* extract administration could produce the antihypertensive activity. In this study, we would like to determine whether coadministration of *H. sabdariffa* extract with captopril which is given in the same manner in a pharmacodynamics study could produce pharmacokinetic herb-drug interaction.

Captopril is an unstable and photolabile compound with a sulfhydryl group prone to dimerization and binding to various endogenous plasma molecules with sulfhydryl groups such as cysteine and glutathione [21, 22]. In this experiment, we used 2-4-dibromoacetophenone as a derivatization agent to prevent the sulfhydryl group in captopril from binding to plasma sulfhydryl groups. Ammonia was used during derivatization since the reaction is optimal at basic pH. The reaction was then stopped by adding formic acid [17].

The chromatography system yielded sharp chromatograms for both derivate captopril and propranolol (the IS) within 5 min and a captopril LLOQ of 3 ng/mL. The accuracy and precision of the captopril measurements were determined by five replicates of each quality control sample (3, 9, 40, and 80 ng/mL), which yielded %CV values of 1.56%–14.38% and % Diff values of less than 15%. Thus, the chromatographic method was of sufficient reliability and reproducibility for captopril measurement within the analytical range [23]. The selectivity, carry over, and matrix effect tests also showed that the chromatographic method fulfills the acceptance criteria. Therefore, the developed method could be concluded as a valid method.

It is known that captopril absorption by the gut is influenced by the presence of food [21, 22]. Therefore, captopril was administered one hour after the final oral administration of *H. sabdariffa* L. However, the present study showed that two weeks of regular *H. sabdariffa* L. aqueous extract consumption reduced the AUC and peak plasma concentration of captopril suggesting reduced efficacy (Tables 4 and 5) and the geometric mean ratios were falling outside the bioequivalent range. There is no side effect observed after the treatment of captopril and *Hibiscus sabdariffa* aqueous extract. Previous herb-drug pharmacokinetic interaction studies on *Hibiscus sabdariffa* aqueous extract showed similar observation. Coadministration of the extract could increase the clearance of acetaminophen, reduce the AUC and *C*max of chloroquine, and increase the clearance as well as reduce the AUC of simvastatin [4, 7, 8]. However, the coadministration of *Hibiscus sabdariffa* aqueous extract with captopril does not influence the antihypertensive potency of captopril or its effects on biomarkers of the renin-angiotensin-aldosterone system (RAAS). The coadministration also does not negatively influence captopril action [13].

Comparing the results of this study and our previous study [13], we could not determine the exact mechanism of this herb-drug interaction. However, there are some hypotheses which could explain the interaction as follows: *H. sabdariffa* L. extract has well documented antioxidant activity, which could increase the plasma concentration of GSH [24]. This could in turn increase the formation of a mixed disulfide captopril complex and lower the concentration of free captopril in plasma [25]. Quercetin contained in *Hibiscus sabdariffa* aqueous extract could reduce the expression of the PEPT1 transporter. The transporter plays an important role in absorption of ACE inhibitor drugs, such as captopril. It therefore could reduce the concentration of captopril absorbed into blood circulation and finally reduce the concentration of free captopril in plasma [26, 27]. Two weeks of coadministration of *H. sabdariffa* extract is sufficient enough to affect the GSH level or the expression of the PEPT1 transporter.

The total number of samples used in this study is limited; therefore, there is a possibility that the effect shown in humans especially when performed in large population will be different. In order to minimize the differences possibility, the doses used in this study were extrapolated from the usual doses given to humans. The researcher also applied the refinement procedure during housing and blood collection as explained in the Materials and Methods section to minimize any bias during the study.

## 4. Conclusion

Two weeks of coadministration of *H. sabdariffa* L. extract prior to administration of captopril could affect the pharmacokinetic profile of captopril significantly Therefore, regular consumption of *H. sabdariffa* L. aqueous extracts such as tea should be avoided when taking captopril.

## Figures and Tables

**Table 1:** Gradient profile of the mobile phase used to analyze plasma captopril.

Time (min)	Flow rate (mL/minutes)	0.1% Formic acid in aquadest (%)	Acetonitrile (%)
0	0.2	60	40
1	0.2	10	90
2	0.2	10	90
2.5	0.2	60	40
5	0.2	60	40

**Table 2:** Operational parameters for mass spectrometry detection of captopril and propranolol.

Parameter	Value
Cone (V)	Captopril, 32; propranolol, 22
Collision energy (V)	Captopril, 36; propranolol, 18
Mode of analysis	Positive
Ion transition for captopril (Da)	m/z 414.01/216.082
Ion transition for propranolol (IS) (Da)	m/z 260.16/183.006

**Table 3:** Accuracy and precision of plasma captopril detection.

Actual concentration (ng/mL)	Mean measured concentration ± standard deviation (ng/mL)	% CV	% Diff
3	3.05 ± 0.44	14.38	12.65
9	9.65 ± 1.00	10.38	12.08
40	38.74 ± 4.14	10.69	9.51
80	76.36 ± 1.19	1.56	4.54

**Table 4:** Plasma pharmacokinetic profile of captopril and its coadministration with *Hibiscus sabdariffa* aqueous extract in rats.

Parameter	Group I (*n* = 6)	Group II (*n* = 6)
AUC_0−t_ (ng h/mL)	956.86 ± 314.28	162.35 ± 41.00^*∗*^
AUC_0−∞_ (ng h/mL)	993.32 ± 339.55	166.73 ± 41.82^*∗*^
*C*_max_ (ng/mL)	282.98 ± 124.79	60.02 ± 28.84^*∗*^
*T*_max_ (h)	2.17 ± 1.47	1.46 ± 0.87
*T*_1/2_ (h)	1.51 ± 0.52	1.58 ± 0.25
Cl/F (mL/h·kg)	0.0257 ± 0.0115	0.1418 ± 0.0338^*∗*^
Vd/F (mL/kg)	0.0541 ± 0.0226	0.3205 ± 0.0790^*∗*^
Ke (1/h)	0.4907 ± 0.1149	0.4475 ± 0.0597

Group I, captopril alone; group II, coadministration with *Hibiscus sabdariffa* extract; AUC, area under the curve; *C*max, maximum concentration; Cl/F, apparent total body clearance; Vd/F, apparent volume of distribution. ^*∗*^*P* < 0.05.

**Table 5:** 90% CI for geometric mean ratios of AUC_0−t_, AUC_0−∞,_ and *C*_max_.

Parameter	Geometric mean		Geometric mean ratio^*∗*^	90% confidence interval^+^
	Group I	Group II		
AUC_0−t_ (ng h/mL)	907.1936	158.3434	0.1745	0.1254–0.2429
AUC_0−∞_ (ng h/mL)	937.3462	162.5549	0.1734	0.1232–0.2442
*C*_max_ (ng/mL)	260.6754	55.2459	0.2119	0.1337–0.3359

Group I, captopril alone; group II, coadministration with *Hibiscus sabdariffa* extract; AUC, area under the curve; *C*max, maximum concentration; ^*∗*^Group II/group I; ^+^Lower limit/upper limit.

## Data Availability

The data used to support the findings of this study are available from the corresponding author upon request.

## Abbreviations:

ACE: Angiotensin converting enzyme
ACN: Acetonitril
ANOVA: Analysis of variance
AUC: Area under curve
BEH: Ethylene bridged hybrid
BW: Body weight
Cl/F: Th apparent total body clearance
*C*_max_: Maximum concentration
CV: Coefficient of variation
DAD: Diode-array detector
EDTA: Ethylenediaminetetraacetic acid
GSH: Reduced glutathione
HS: *Hibiscus sabdariffa*
HCA: Hidroxycitric acid
HPLC: High-pressure performance chromatography
IS: Internal standard
Ke: The values of elimination constant
LLOQ: Lower limit of quantification
PEPT1: Peptide transporter 1
p.o.: Per-oral administration
QCL: Quality control sample low concentration
QCM: Quality control sample medium concentration
QCH: Quality control sample high concentration
SD: Standard deviation
*T*_max_: Maximum time
T_1/2_: Half-life;
UPLC/MS-MS: Ultrahigh-pressure liquid chromatography-tandem mass spectrometry
TQD: Triple quadrupole detector
Vd/F: The apparent volume of distribution.
